# Agrimonolide and Desmethylagrimonolide Induced HO-1 Expression in HepG2 Cells through Nrf2-Transduction and p38 Inactivation

**DOI:** 10.3389/fphar.2016.00513

**Published:** 2017-01-11

**Authors:** Lei Chen, Hui Teng, Kalin Yanbo Zhang, Krystyna Skalicka-Woźniak, Milen I. Georgiev, Jianbo Xiao

**Affiliations:** ^1^College of Food Science, Fujian Agriculture and Forestry UniversityFuzhou, China; ^2^School of Chinese Medicine, The University of Hong KongHong Kong, Hong Kong; ^3^Department of Pharmacognosy with Medicinal Plant Unit, Medical University of LublinLublin, Poland; ^4^Group of Plant Cell Biotechnology and Metabolomics, The Stephan Angeloff Institute of Microbiology, Bulgarian Academy of SciencesPlovdiv, Bulgaria; ^5^State Key Laboratory of Quality Research in Chinese Medicine, Institute of Chinese Medical Sciences, University of MacauTaipa, Macau

**Keywords:** agrimonolide, desmethylagrimonolide, HepG2 cells, Nrf2, heme oxygenase-1, p38 MAPK signaling pathway

## Abstract

Agrimonolide and desmethylagrimonolide are the main bioactive polyphenols in agrimony with well-documented antioxidant, anti-diabetic, and anti-inflammatory potential. We report here for the first time that agrimonolide and desmethylagrimonolide stimulate the expression of phase II detoxifying enzymes through the Nrf2-dependent signaling pathway. Agrimonolide and desmethylagrimonolide also possess considerable protective activity from oxidative DNA damage. In order to explore the cytoprotective potential of agrimonolide and desmethylagrimonolide on oxidative stress in liver, we developed an oxidative stress model in HepG2 cells, and check the hypothesis whether Nrf2 pathway is involved. Western blotting and luciferase assay revealed that exposure of HepG2 cells to agrimonolide or desmethylagrimonolide leads to increased heme oxygenase-1 (HO-1) expression by activating ARE through induction of Nrf2 and suppression of Kelch-like ECH-associated protein 1 (Keap1). Moreover, agrimonolide and desmethylagrimonolide also activated ERK signaling pathways and significantly attenuated individual p38 MAPK expression, subsequently leading to Nrf2 nuclear translocation. In conclusion, our results indicated that transcriptional activation of Nrf2/ARE is critical in agrimonolide and desmethylagrimonolide-mediated HO-1 induction, which can be regulated partially by the blockade of p38 MAPK signaling pathway and inhibiting nuclear translocation of Nrf2.

## Introduction

Reactive oxygen species (ROS) and reactive nitrogen species (RNS) are involved in a spectrum of physiological and pathological processes (Medzhitov, 2008). Low physiological levels of ROS regulate cellular signal transduction and play an important role in normal cell proliferation (Scandalios, 2002). High levels of ROS, however lead to apoptosis and necrosis. Evidence has generally suggested that ROS were required for the downstream signaling effects (Forman and Torres, 2002). Similar to the regulation of protein function by phosphorylation, oxidation of cysteine residues by ROS results in conformational, structural, and direct catalytic consequences on the targeted signaling proteins (Cross and Templeton, 2006). However, very few observations of these modifications in response to pharmacologically relevant signaling stimuli have been proven. Oxidative stress is one of the factors in occurrence and development of many diseases (Christen, 2000; Lin and Beal, 2006). Oxidative stress occurs when this critical balance is disrupted due to excess of ROS, antioxidants depletion, or both. To counteract the oxidant effects and restore redox balance, cells must reset important homeostatic parameters. In living cells, the major sources of endogenous ROS are hydrogen peroxide and superoxide anion, which are generated by the production of cellular metabolism such as mitochondrial respiration (Waris and Ahsan, 2006). Unstable mitochondrial membrane potential and redox transitions can occur as a result of diverse pathological states such as ischemia/reperfusion injury and toxin exposure, and can have negative consequences for mitochondrial integrity and cellular survival (Zorov et al., 2006). Alternatively, hydrogen peroxide may be converted into water by the enzymes catalase or glutathione peroxidase (Apel and Hirt, 2004).

The antioxidant responsive element (ARE) is a *cis*-acting regulatory element of genes encoding phase II detoxification enzymes and antioxidant proteins, such as NADPH, quinone oxidoreductase, glutathione-transferases, and glutamate-cysteine ligase. Interestingly, it has been reported that NF-E2-related factor 2 (Nrf2) regulates a wide array of ARE-driven genes in various cell types (Lee and Johnson, 2004). Since Nrf2/ARE signaling pathway is one of the key steps for the cells to resist oxidative stress, it is of great significance to study the role of Nrf2/ARE signaling pathways in the health damage effect caused by H_2_O_2_ and its specific mechanism of action. Under quiescent conditions, the transcription factor Nrf2 interacts with the actin-anchored protein Keap1 mostly localized in the cytoplasm (Kensler et al., 2007). However, importantly, response to oxidative stress and cysteine sensors within Keap1 are oxidized or conjugated, leading to the accumulation of closed conformation of the Keap1-Nrf2 complex (Bertrand et al., 2015). Keap1 interaction with Nrf2 leads to the sequestration of Nrf2 in the cytoplasm and the enhancement of Nrf2 degradation by proteasomes conferring tight regulation in the response (Itoh et al., 2004). Even under oxidative stress conditions, where Nrf2 is liberated from Keap1 repression, Nrf2 is still subjected to proteasome degradation, indicating the existence of Keap1-independent degradation of Nrf2. On another aspect, the prototype coenzymes, such as glutathione S-transferases (GSTs) (Friling et al., 1990) and NAD(P)H-quinone oxidoreductase 1 (NQO1) (Rushmore et al., 1991), are regulated by the Nrf2-ARE signaling pathway, along with a subset of antioxidant genes including heme oxygenase 1 (HO-1) (Alam et al., 1995), the subunits of g-glutamylcysteine synthetase (g-GCS) (Mulcahy et al., 1997), and thioredoxin (Kim et al., 2001). Besides the direct action on anti-oxidative stress, the antioxidant effect of some polyphenols or polyphenol-rich foodstuffs on down-regulation of free radicals have been described in our previous studies as well as other relevant studies (McMahon et al., 2003; Chen and Kang, 2013, 2014a; Chen et al., 2014b).

Polyphenols in plants play a crucial role against ultraviolet light and plant pathogens as they have been proven to have anti-oxidative, anti-microbial and anti-proliferative effects. The beneficial effects of polyphenols were widely investigated in both *in vivo* and *in vitro* conditions and model systems. Nowadays, polyphenols or polyphenol-rich extracts are used as functional ingredients in foods and dietary supplements. Owing to the complexity of polyphenols, various experimental approaches are employed to examine the characterization of the bioactive polyphenol components from herbs. Furthermore, the interactions between polyphenols and other food ingredients, such as protein, fatty acid, and dietary fiber, are investigated as it might affect the bio-accessibility of polyphenols during digestion, absorption, and metabolism in human (Gleichenhagen and Schieber, 2016). The application of polyphenols is recommended to restrict the occurrence of lipoper oxidation and maintain human health (Brenes et al., 2016). Many studies have shown that polyphenols possess antioxidant property, and daily intake of polyphenols enriched diets could prevent relative diseases. Besides, the basic chemical structure of polyphenols has some features in common with polyaromatic hydrocarbons. Thus, polyphenols are found to interact with cellular defense systems such as NQO1 (Favreau and Pickett, 1995), g-GCS (Rushmore et al., 1990), HO-1 (Inamdar et al., 1996), thioredoxin (Ren and Smith, 1995), and inducible nitric oxide synthase (Kuo et al., 2000). Although, many polyphenol compounds could induce ARE activation, and some of them enhance Nrf2 expression or nuclear translocation, but the precise mechanism to explain why these polyphenols influence the Nrf2-Keap1 complex to activate ARE is poorly understood.

**Figure 1:**
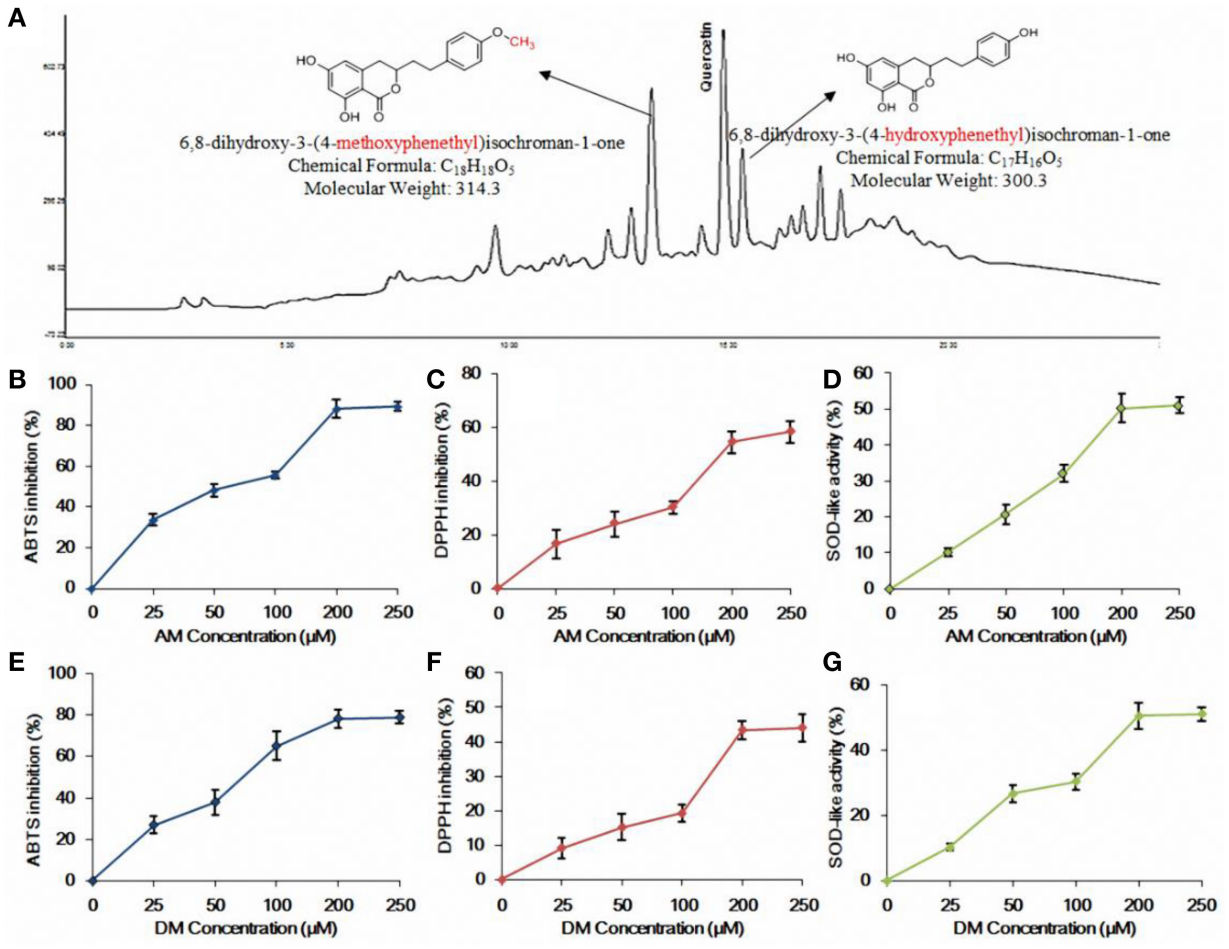
**HPLC profile and ***in vitro*** antioxidant activity of AM and DM. (A)** HPLC profile of the methanolic extract of agrimony and structures of AM and DM; **(B)** ABTS radical scavenging effect of AM; **(C)** DPPH radical scavenging effect of AM; **(D)** SOD-like activity of AM; **(E)** ABTS radical scavenging effect of DM; **(F)** DPPH radical scavenging effect of DM; **(G)** SOD-like activity of DM. Values of each curve are mean values ± SD (*n* = 3).

Agrimony (*Agrimonia pilosa* Ledeb., Xianhecao in Chinese), belonging to the Rosaceae family, is known as a traditional Chinese medicine. It has been reported that agrimony can help in treating chronic prostatitis, but the active principles in agrimony for this effect have not been clearly reported yet. Natural products present a key role in drug discovery programs. Consequently, a large number of compounds have been investigated in our previous studies. We have recently isolated symbolical compounds from agrimony by utilizing antioxidant activity-guided fractionation (Huang et al., 2015). It was found that agrimonolide (AM) and desmethylagrimonilide (DM) (Figure 1) were highly presented in this Chinese edible herb, showing a powerful antioxidant activity (Huang et al., 2015) and a strong anti-diabetic potential (Chen and Kang, 2014b), and hence contributing to restore the diminished AMP-activated protein kinase (p-AMPK) levels in high-glucose-incubated adipocyte (Chen and Kang, 2014b). Therefore, the aim of the present work was to investigate compare the antioxidant activities and superoxide dismutase (SOD)-like effect in regard to underlying antioxidant and hepatoprotective mechanism of AM and DM. We examined the requirement of contiguous phosphoserine residues in antioxidative stress activity using an *in vitro* model of H_2_O_2_-induced oxidative stress in intestinal epithelial cells, and elucidated potential signaling mechanisms by quantitative RT-PCR array. Furthermore, the role of Nrf2 and associated mitogen-activated protein kinases (MAPKs) involved in Nrf2 activation in the antioxidative stress activity of phosphoserine dimers was demonstrated.

## Materials and methods

### Materials and chemicals

Both AM and DM were previously isolated from *A. pilosa* (Huang et al., 2015). The purities of AM and DM (95%) were further confirmed by using NMR and MS spectra. NMR spectra were recorded on a Bruker 400 Ultra Shield NMR spectrometer (^1^H 400 MHz; ^13^C 100.61 MHz). Low resolution mass spectrometry was taken on a Triple Quadrupole mass spectrometer (Micromass Quattro Micro API, Waters). Column chromatography was carried out on SiO_2_ (Merck 9385) or a Combiflash system (Teledyne-Isco; Lincoln, NE, USA) using SiO_2_ or C_18_ prepacked columns. TLC was performed on precoated SiO_2_ F_254_ plates (Merck 5554 and 5744) and visualized under UV light and by spraying with H_2_SO_4_-MeOH (1:1) followed by heating. HPLC was carried out on a Merck-Hitachi instrument, with UV detection (254 nm). Tested compounds were purified to ≥ 95% HPLC (Merck LiChrospher 100 RP-18; 5 μm, 125 × 4 mm column). The antibodies and horseradish peroxidase conjugated anti-goat secondary antibody were purchased from Santa Cruz Biotechnology (Santa Cruz, CA, USA).

### DPPH radical inhibition

1,1-Diphenyl-2-picrylhydrazyl (DPPH) radical inhibition assay was completed by following our previous study (Gleichenhagen and Schieber, 2016). A volume of 10 μL of sample was added to 190 μL solution of 0.4 mM DPPH dispersed in 95% ethanol. The sample was shaken vigorously and kept in the dark at room temperature for 30 min. Control was prepared using pure ethanol. The absorbance was measured at 517 nm.

### ABTS radical inhibition

2, 2′-Azino-bis-(3-ethylbenzothiazoline-6-sulphonic acid) (ABTS) scavenging activity assay was completed using the colorimetric method (Brenes et al., 2016). ABTS radical cation was prepared by mixing 5 mL of 7 mM ABTS solution with 45 mL of 2.45 mM K_2_S_2_O_4_ solution. This mixture was stored in the dark at room temperature for 12–16 h before test. Prior to the assay, this mixture is diluted with 80% ethanol at an approximate ratio of 1:45 and adjusted to yield an absorbance of 0.70 ± 0.05 at 734 nm. A volume of 190 μL ABTS solution was added to 10 μL of sample and the mixture was vortexed for 1 min. After incubation at room temperature for 6 min the absorbance was measured.

### SOD-like activity

This assay was performed according to our previous study (Chen and Kang, 2014b). Each sample or ascorbic acid (positive control) was mixed with Tris-hydrochloric acid buffers (50 mM Tris-aminomethane+10 mM EDTA, pH 8.5) and 7.2 mM pyrogallol. The reaction was stopped by the addition of HCl solution. Absorbance of the mixture was determined at 420 nm against a blank, and this assay was replicated for 3 times.

### Cell culture and viability

Human HepG2 cells were obtained from GibcoBRL (now Invitrogen Corporation, Carlsbad, CA, USA), and cultured in Dulbecco's modified Eagle's medium (DMEM) containing 10% FBS and 1% penicillin streptomycin under 5% CO_2_ atmosphere at 37°C. Compounds' stock solutions were prepared in DMSO, and the final concentration in cell culture ranged from 25 to 150 μM. Hydrogen peroxide (H_2_O_2_) with a concentration of 1 mM was used for stimulation. 3-(4, 5-Dimethylthiazol-2-yl)-2, 5-diphenylt etrazolium bromide (MTT) assay was used to determine the cell viability (Chen et al., 2014a). Cells were plated at a density of 1 × 10^5^ cells/well in a 12-well plate overnight and incubated with tested compounds for 4 h and then treated with 1 mM hydrogen peroxide (H_2_O_2_) for 24 h. MTT working solution of 0.3 mg/mL final concentration (Promega, CA, USA) was added to the culture plates and after reacting for 2 h. MTT formazan products were solubilized with DMSO on a shaker platform. Finally, the absorbance was measured at 450 nm using Fluorescence spectrophotometer SpectraMax M5 (Molecular Devices, CA, USA). The results were expressed as percentage of the control.

### Flow cytometer analysis

For detection of cytotoxic drug-induced apoptosis, a FITC Annexin V apoptosis detection kit (BD Pharmingen™, BD Biosciences) was used. Briefly, 6 × 10^4^ cell/mL of HepG2 cells were seeded in six-well plates in complete medium and allowed to attach for 24 h. On the next day, the medium was discarded and new medium, with 30 μM of the tested compounds added. N-acetylcysteine (NAC) was used as positive control (1.0 mM). Cells were further incubated for 72 h, in 5% CO_2_ at 37°C. After the incubation period, cells were trypsinized, washed in PBS and stained according to kit manufacturer's instructions. Stained cells were analyzed using BD Accuri C6 flow cytometer (BD Pharmingen™, BD Biosciences) and data was processed with BD Accuri C6 software. Each sample was assessed using a collection of 10,000 events. The mean values and standard deviations were calculated from three independent experiments.

### Intracellular ROS level determination

Intracellular ROS production in RAW 264.7 treated with hydrogen peroxide (H_2_O_2_) was monitored by flow cytometry according to dichlorofluorescein assay (Kim et al., 2009). 2′, 7′-dichlorofluorescein-diacetate (DCF-DA) can be deacetylated in cells, where it can react quantitatively with intracellular radicals to convert into its fluorescent product. Hydrogen peroxide or low-molecular weight phydroeroxides produced by cells oxidize DCHF to the highly fluorescent compound, 20, 70-dichloro-fluorescein (DCF). Therefore, DCF-DA was used to evaluate the generation of ROS in oxidative stress. In the present study, cells were plated at a concentration of 4 × 10^5^ cells/well in 12-well plates overnight, and then treated with AM and DM for 24 h in the present of H_2_O_2_. Later, the compound-treated cells were washed twice with cold-PBS to remove the extracellular compounds, and the fluorescence intensities of the stained cells were determined under absorbances of 485 and 535 nm (excitation and emission wavelengths, respectively) using a fluorescence spectrophotometer (SpectraMax M5, Molecular Devices, CA, USA).

### Determination of radical mediated DNA damage

Hydrogen peroxide induced DNA oxidation was examined, following a protocol described elsewhere (Ballinger et al., 2000). Briefly, 100 μL of DNA reaction mixture was prepared by adding pre-determined concentrations of test sample (or same volume of distilled water as a control), 200 μM final concentration of FeSO_4_, 1.0 mM final concentration of H_2_O_2_ and 50 μg/mL final concentration of genomic DNA in the same order. Then, the mixture was incubated at room temperature for 30 min and reaction was terminated by adding 10 mM final concentration of EDTA. 20.0 μL of reaction mixture containing about 1.0 μg of DNA were electrophoresed with 0.5% low-melting-point agarose (Promega Co., Madison, WI, USA). Gels were then stained with 1 mg/mL ethidium bromide and visualized by UV light using AlphaEase gel image analysis software (Alpha Innotech, CA, USA).

### Western blotting

The cells were harvested, washed twice with PBS buffer, and lysised in RIPA buffer consisting of 20 mM Tris-HCl pH 7.5, 150 mM NaCl, 1 mM EDTA, 1 mM EGTA, 1% NP-40, 1% sodium deoxycholate, 2.5 mM sodium pyrophosphate, 1 mM β-glycerophosphate, 1 mM Na_3_VO_4_, and 1 μg/mL leupeptin (Cell Signaling Technology; Beverly, MA, USA). Eluted protein concentration was measured using the DC protein assay (Bio-Rad, Richmond, CA, USA) according to the manufacturer's instructions. For the western blot assay, equal amounts of extracted proteins were separated by SDS-polyacrylamide gel electrophoresis (PAGE) and transferred to nitrocellulose membranes by semi-dry electro-blotting (Bio-Rad). Proteins that were extracted from either whole cell lysate or nuclei were separated by SDS-PAGE, transferred to nitrocellulose membranes, and detected with an Nrf2 antibody. Membrane was washed three times for 15 min with washing solution of PBS containing 0.05% Tween-20. After washing, the blots were incubated with the secondary antibody for 2 h. Then, it was washed three times with PBS washing solution, and protein bands were detected using enhanced chemiluminesence (ECL) system and visualized by chemiluminescent light. Immunoblots for phosphorylation of ERKs, JNKs, and p38 kinase were carried out as described in the protocol of the manufacturer, using phosphorylated antibodies against phosphorylated sites of ERKs, JNK, and p38 kinase. Non-phospho specific antibodies against ERKs and p38 kinase proteins provided by the assay kits were used to normalize the phosphorylation assay with the same transferred membrane blot.

### Statistical analysis

The data were expressed as the mean ± SD. The data were analyzed by one-way ANOVA or two-way ANOVA (Tukey's test) using the IBM SPSS Statistics 20.0 for Mac (IBM Corporation, Armonk, NY). The Kolmogorov–Smirnov and the Levene's tests were used to examine the normality and equality, respectively. The differences between the mean values were considered to be statistically significant when *p* ≤ 0.05.

## Results

### AM and DM showed strong antioxidant activity

Both AM and DM, from *A*. *pilosa*, tested in this study were preliminarily and systematically identified using RP-HPLC with UV detector by comparing with authentic compounds isolated previously (Huang et al., 2015). AM and DM were found as effective scavengers for DPPH and ABTS free radicals. At 200 μM concentration level, both of AM (Figure 1B) and DM (Figure 1E) showed that the ABTS radicals scavenging activity reached plateau of 80%. There was no significant variation of DPPH inhibitory effect between AM (58.2%, Figure 1C) and DM (44.8%, Figure 1F), at the same concentration. Scavenging of DPPH radicals allows the evaluation of hydrogen-donating potency of these two compounds. The SOD-like activity of AM and DM, at concentration of 200 μM, were 50.7 and 51.2% respectively. No significant change of antioxidant effect was observed when comparing concentration range of 200–250 μM for both of AM (Figure 1D) and DM (Figure 1G). Based on these findings, subsequent studies were conducted at the concentrations of AM and DM ranging from 25 to 200 μM.

### Effects of AM and DM on HepG2 cells

**Figure 2:**
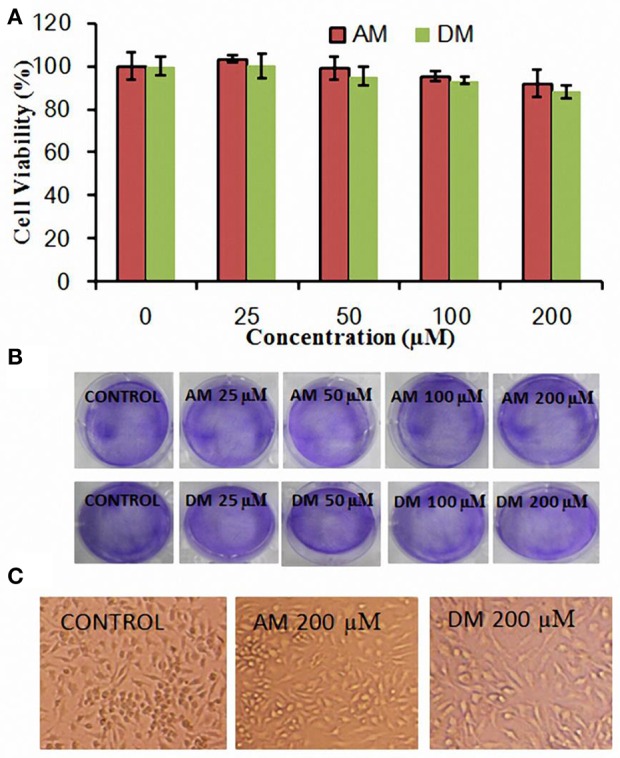
**Cytotoxicity of AM and DM in HepG2 cells. (A)** Cells were plated at a density of 1 × 10^5^ cells/well in 12-well plate overnight and incubated with tested compounds for 4 h and viability was measured by MTT assay. The values for each compound concentration tested represent the average (mean ± SD; *n* = 3); **(B)** Cellular morphology differences by addition of various concentration of AM and DM; **(C)** Histopathological changes in HepG2 cells after AM and DM treatment at the concentration of 200 μM.

The growth of HepG2 cells in the presence of various concentrations of AM and DM was examined by using MTT assay. As shown in Figure 2A, AM and DM treatment did not cause significant change in MTT based cell viability, compared to those of the untreated control cells. Typical microscopic images of AM and DM extracts on human tumor cells are shown in Figure 2B. Compared to the control, the size as well as the shape of all cells were not significantly changed after treatment with AM or DM. Similar results were found in Figure 2C, cells shrinking and falling off from the adherent state were not observed even at the highest concentrations tested.

### AM and DM regulated intracellular ROS in HepG2 cells

**Figure 3:**
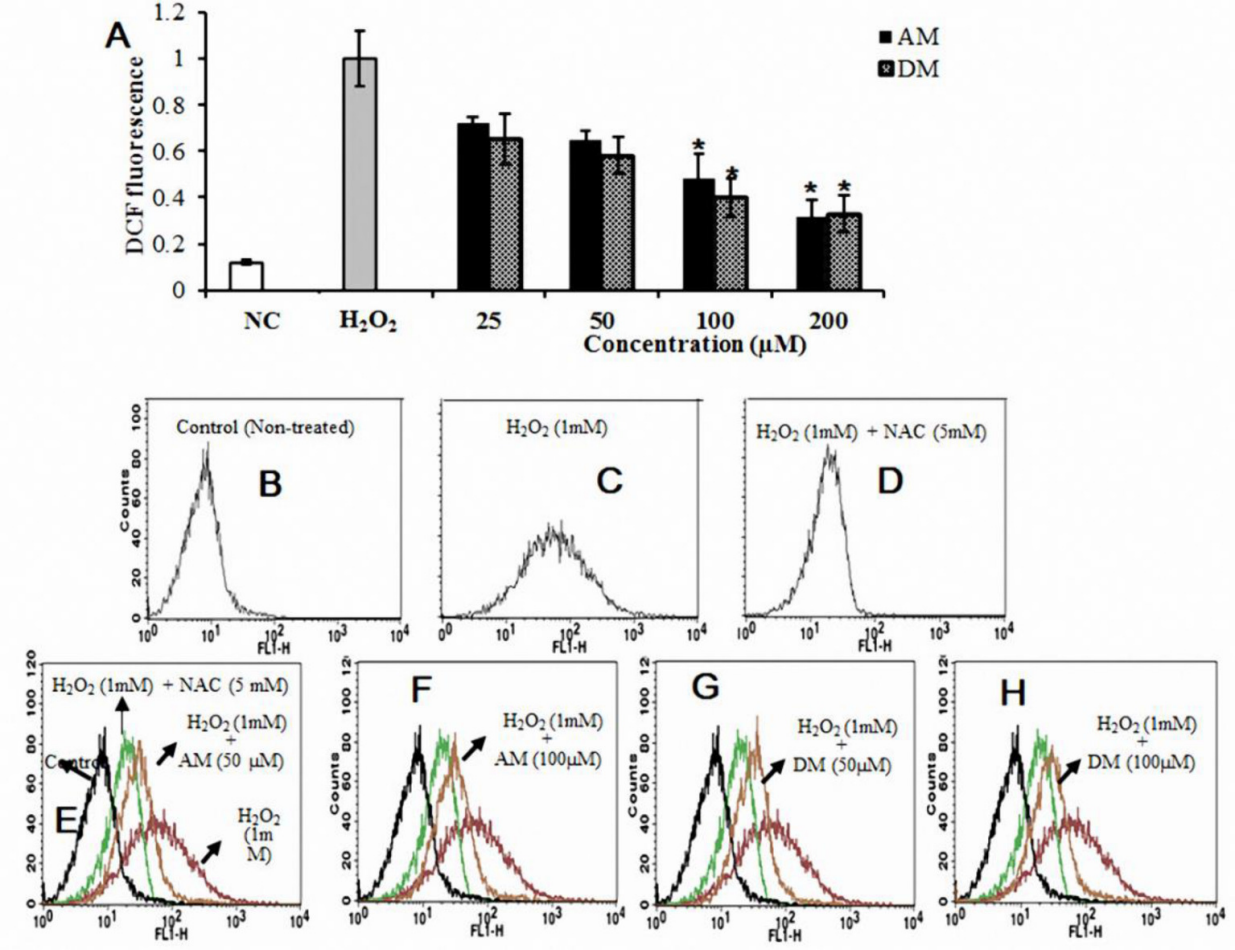
**Cellular antioxidant effects of AM and DM. (A)** Intercellular ROS levels and **(B)** estimated by 2′, 7′-dichlorofluorescein-diacetate (DCF-DA) by using fluorescence spectrophotometer; **(C)** Decreases in relative fluorescence is reflected with a leftwards shift in x-axis in line histograms compared to H_2_O_2_-induced cells. Cells exposed to 1 mM NAC plus 1 mM H_2_O_2_ for 24 h **(D)**, A trend of in peak movement from left to right was found after the addition of 50 or 100 μM AM and DM **(E-H)**. One-way arrow indicates the shift from right (fluorescent cells) to left (non-fluorescent cells) values of each column are mean values ± SD (*n* = 3). ^*^p < 0.05 indicate significant differences compared with the control group.

It has been reported that the ROS can contribute to mitochondrial damage, which may facilitate further release of ROS into the cytoplasm (Simon et al., 2000). To investigate the effect of AM and DM (from 50 to 200 μM) on the generation of intracellular ROS, cells were pretreated with the samples and then incubated with 1 mM H_2_O_2_ (Figure 3A). As expected, pre-treatment of cells with AM and DM also blocked the increase of intracellular ROS generation. Both of them lead to reduction of ROS in H_2_O_2_-induced HepG2 cells: from 36 to 48% for AM (50–200 μM), and from 60 to 66% for DM, at the same concentration range. The level of intracellular ROS was monitored by flow cytometry using a peroxide-sensitive fluorescent probe, DCFDA (2′,7′-dichlorofluorescein diacetate). Fluorescence intensity was directly proportional to the level of dichlorofluorescein (DCF) formed intracellularly, which can be easily detected by flow cytometry. In the presence of intracellular ROS, DCF-DA was oxidized into fluorescent 2′, 7′-dichlorofluorescein (H_2_DCF). In flow cytometry analysis, an increase of intracellular ROS level was noticed in H_2_O_2_-induced cells_,_as demonstrated by the shift of histogram of fluorescence from left (Figure 3B) to the right panel (Figure 3C). To elucidate the role of ROS in H_2_O_2_ induced apoptosis, the antioxidant of N-acetylcysteine (NAC) was used, which has been shown previously to be a radical scavenger functioning as both redox buffer and reactive oxygen intermediate scavenger. Cells exposed to 1 mM NAC plus 1 mM H_2_O_2_ for 24 h did not exhibit the characteristic features of cell shrinking, rounding, and partial detachment as cells exposed to H_2_O_2_ alone (Figure 3D). However, when cells were pre-treated with NAC, the fluorescence were decreased to 3.0-fold, and the distance of peak movement from left to right was observed (Figure 3E) as compared with H_2_O_2_ stimulated cells. A same trend of in peak movement was found after the addition of 50 or 100 μM AM and DM (Figures 3E–H).

### AM and DM inhibit DNA oxidation

**Figure 4:**
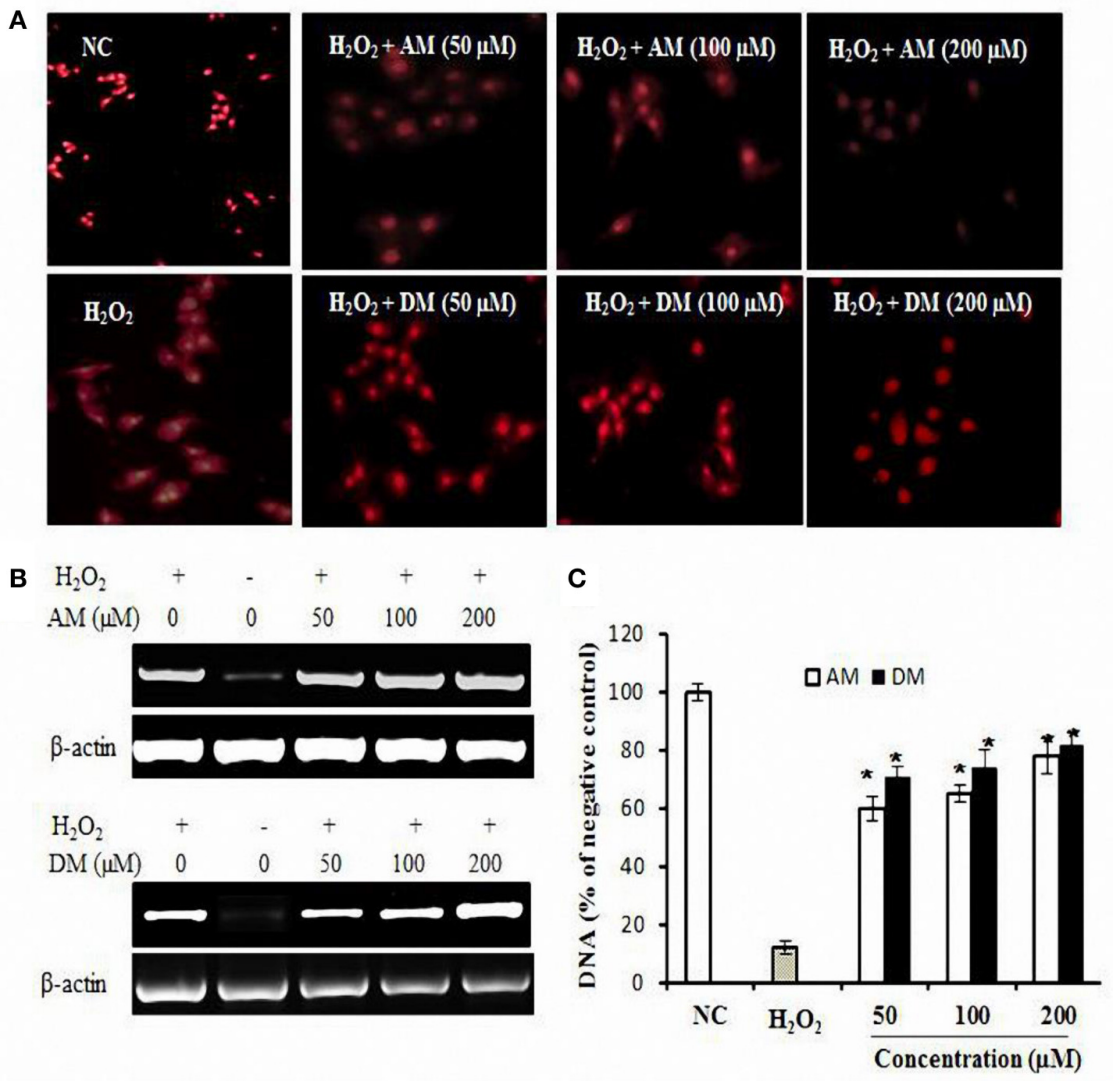
**DNA oxidative protection by AM and DM in HepG2 cells. (A)** The fluorescence images of HepG2 cells treated with H_2_O_2_ only or along with samples and a nuclear localizing propidium iodide (PI) dye. Cells were treated with 1 mM H_2_O_2_ and then washed and stained with 10 μg/ml PI. Fluorescence images were immediately captured by Carl Zeiss fluorescence microscope using PI fluorescence λ 575 nm; **(B)** Extracted DNA levels were measured by RT-PCR; **(C)** AM and DM dose-dependent oxidative protection. Values of each column are mean values ± SD (*n* = 3). ^*^p < 0.05 indicate significant differences compared with the control group.

Hydrogen peroxide as a mediator of cytotoxicity could cause most of the oxidative damage in biological systems by the reaction between H_2_O_2_ and the presence of metal ions (Nathan et al., 1979). The comet assay was used to detect cellular DNA damage (Olive and Banáth, 2006), since DNA within the nucleoid from each cell typically appears as either an intact spherical mass (i.e., no DNA damage) or as a “comet” (i.e., DNA damage) upon staining with ethidium bromide, visualized by fluorescence microscopy. A basic assumption is that the overall span of tail region of the comets is a general indication for the extent of DNA single-strand breakage (Kelly et al., 2001). The photographs shown in Figure 4 are representative views of the slides for untreated and treated cells. The number and overall tail spans of the comets were found to be consistently similar in viewing different areas of each slide upon replicating the experiments three times and overall comet scores generated reflecting the relative extents of DNA damage. As shown in Figure 4A, cells treated with H_2_O_2_ were characterized by clearly discernible comets compared to control cells. Pre-incubated cells with AM concentration as low as 50 αM resulted in a slight reduction of the overall comet tail spans. Moreover, 100 and 200 αM concentration of AM had additional protective effects against DNA damage. In the case of DM, is also worth of expecting that DM would be able to inhibit DNA damage induced by H_2_O_2_ even at low concentration doses supplied.

In addition to detection of oxidative DNA damage, the extracted DNA was subjected to PCR. As shown in Figure 4B, H_2_O_2_ treatment could induce DNA damage. This damage can be reduced in the presence of AM and DM. The addition of DM at concentration of 200 μM, to the reaction mixture, induced the significant protection to the damage. Higher concentrations of AM were also checked for the dose dependent activity, however there was no difference between each other. Densitometric analysis (Figure 3C) revealed that both of AM and DM significantly decreased the H_2_O_2_-induced formation of DNA strand breaks. No differences could be observed between pre-incubation with the AM and DM. For AM, the protective effect was significantly increased by 57% when the dose increased from 0 to 100 μM. Higher concentrations of AM were also checked for the dose dependent activity but no higher protection at 200 μM concentration was observed. Similar trend of protective effect was found by adding DM into the reaction mixture of H_2_O_2_ induced HepG2 cells. These results related to the simultaneous incubation of the cells with AM and DM were clearly demonstrated by the comets representative mages for each condition.

### AM and DM increased Nrf2 transcription and induced HO-1 expression

**Figure 5:**
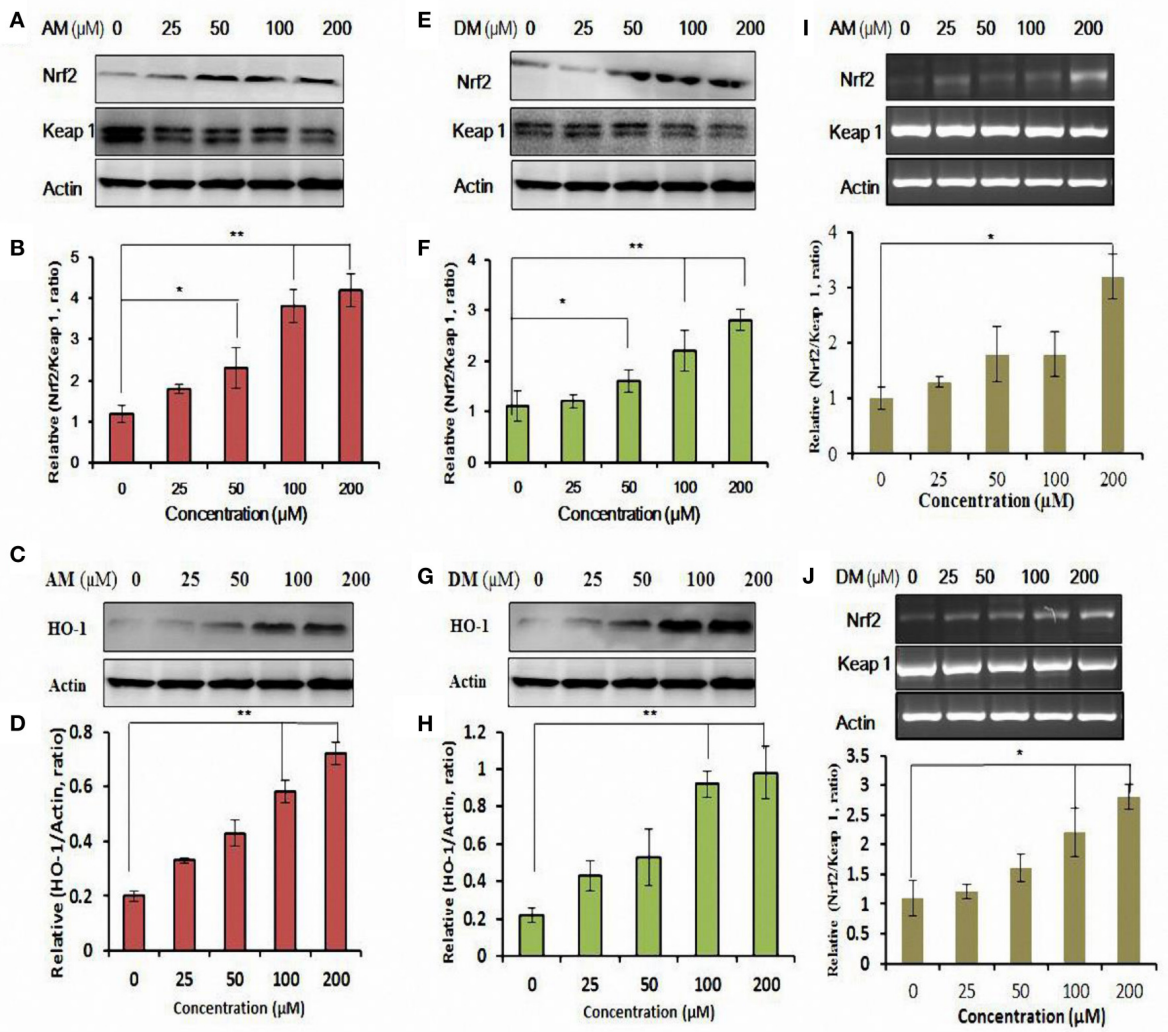
**Functional characterization of AM and DM-mediated expression of Nrf2 and activation of antioxidant response element (ARE) pathway**. Whole cell lysates were harvested and analyzed with antibodies to Nrf2-Keap1 by addition of AM **(A,E)**. **(B,F)** Quantification analysis of the immunofluorescence results of Nrf2/Keap 1 ratio. HO-1 expression was analyzed by Western blotting in cultured HepG2 cells. Cells were treated with various concentrations of AM **(C)** and DM **(G)** for 48 h. The test was repeated three times, and representative blots are shown. Dose-dependent induction of HO-1 protein by AM **(D)** and DM **(H)**. Gene expression of Nrf2/Keap1 affected by AM **(I)** and DM **(J)**. Data shown are representative of three independent experiments. Values of each column are mean values ± SD (*n* = 3). ^*^p < 0.05 indicate significant differences compared with the control group. ^**^p < 0.01.

Interestingly, AM and DM increased Nrf2 expression levels in a dose-dependent manner, with the maximal expression at 200 μM in HepG2 cells (Figures 5A,E).

The integrity of the cytosolic was confirmed by the analysis of the compartment-specific cytosolic α-tubulin. Besides the changes in nuclear levels of Nrf2, the nuclear content of the Nrf2 inhibitor Keap1 was also modified by AM and DM. The calculated nuclear Keap1 content ratio in AM and DM-treated HepG2 cells remained comparable to control values under all doses, whereas ratios of nuclear Nrf2/Keap1 were increased by 0.6 ± 0.1- and 3.0 ± 0.4-fold, respectively, at 25 and 200 μM AM (Figure 5B). Quantification of three independent western blots showed that in the presence of 25 and 200 μM DM, Nrf2/Keap1 increased 0.2 ± 0.1- and 3.6 ± 0.4-fold, respectively (Figure 5F). HO-1 expression was further measured to determine whether AM and DM exhibits potential antioxidant activity by up-regulating the intracellular phase II enzyme in HepG2 cells. Western blot analysis was performed to detect the expression of HO-1 induced by different concentrations of AM (Figure 5C) and DM (Figure 5G). Treatment of HepG2 also induced a concentration-dependent enhancement in HO-1 protein expression (Figures 5D,H). Quantification of three independent western blots showed that in the presence of 25, 50, 100, and 200 αM AM, HO-1 protein increased 0.12 ± 0.03-, 0.23 ± 0.01-, 0.38 ± 0.04-, and 0.5 ± 0.02-fold, respectively. Besides, DM also caused a significant increase of HO-1 protein expression (Figure 5H). These data indicated that both AM and DM are HO-1 inducers. Meanwhile, gene expression of Nrf2/Keap1analysed by qRT-PCR (Figures 5I,J) also confirmed that the observed effects are at transcriptional level.

### AM and DM reduced p38 protein phosphorylation

**Figure 6:**
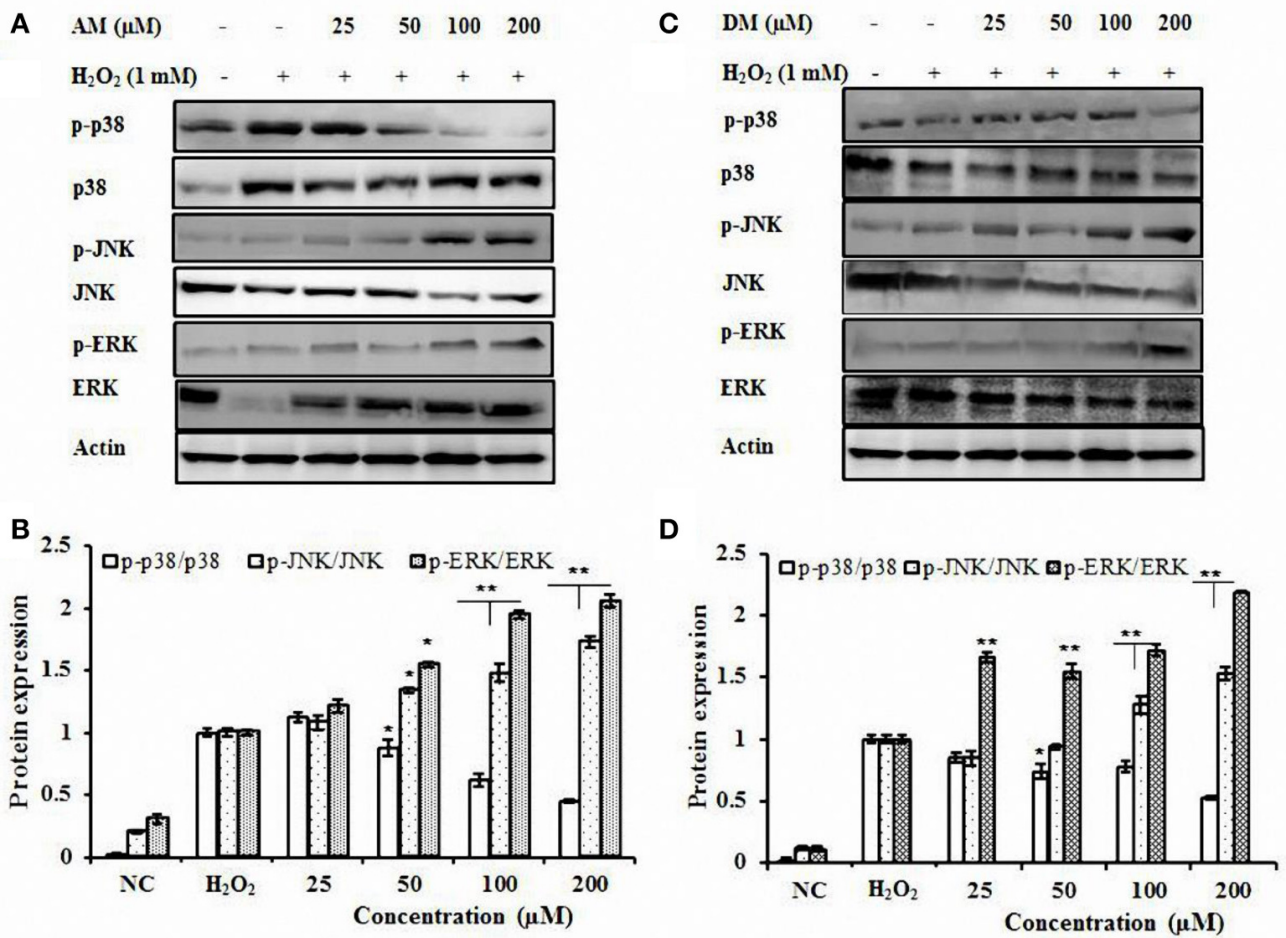
**Effect of AM and DM on p38, ERK, and JNK signaling pathways. (A)** Effect of AM on p38, ERK, and JNK protein expressions; **(B)** AM dose-dependent activation of phosphorylated forms of p38, ERK, and JNK; **(C)** Effect of DM on p38, ERK, and JNK protein expressions. **(D)** DM dose-dependent activation of phosphorylated forms of p38, ERK, and JNK HepG2 cells were treated with AM or DM (NC, control) for 1 h, respectively. The test was repeated three times, and representative blots are shown. ^*^p < 0.01; *p*-value compared with only DHQ-treated cells. ^**^p < 0.01.

To determine the possible role, if any, of MAPKs in H_2_O_2_-mediated HO-1 gene activation, the effect of AM and DM on MAPK activities were examined (Figure 6). Firstly, the effect of AM and DM on three MAPK cascades leading to activation of ERK, p38 and JNK on the survival pathway was analyzed. The activation of these pathways was analyzed with activation-specific antibodies that selectively recognized the active and phosphorylated forms of ERK, p38, and JNK. HepG2 cells treated with H_2_O_2_ alone, total JNK, ERK, and β-actin were not affected but phosphorylations of JNK and ERK were up-regulated by AM and DM treatment. In contrast, AM and DM decreased the phosphorylated levels of p38 in a dose-dependent activation in HepG2. Reduced phosphorylation of p38 (approximately two-fold) was detected after treatment with AM or DM.

## Discussion

Accumulated evidences indicated the biomedical importance of polyphenols as antioxidants. In the present study, we demonstrated that AM and DM, which were isolated from *A. pilosa*, possessed significant antioxidant properties. Different methods were used to evaluate their antioxidant properties, including DPPH, ABTS radical inhibition and SOD-like activities. The radical scavenging assay was based on the measurement of the scavenging ability of antioxidants toward the stable radicals (ABTS and DPPH). Besides, the data on the SOD-like activity obtained were dependent on the antioxidant properties *in vitro*. The main function of SOD is pivotal in ROS release during oxidative stress by ischemia-reperfusion injury, particularly in the myocardium as a part of heart attack. Previously, numerous phenolic products have been found to exhibit SOD-like activity *in vitro* (Chen et al., 2012; Chen and Kang, 2013). ROS and radical species are able to induce DNA damages, which include strand breaks and DNA-protein cross-link (Fong, 2016) among others. In this study, genomic DNA was isolated from HepG2 cells in order to examine the protective effect of the AM and DM against DNA oxidative damage induced by H_2_O_2_ stimulation. DNA damage only occurred in the control group combined with H_2_O_2_, indicating that oxidative stress induced apoptosis (Figure 3). However, the presence of AM and DM exhibited in a clear dose-dependent inhibition effect against DNA oxidation and elevated concentrations well suppressed DNA oxidative damage (Figure 4). The results of the DNA nicking assays showed that AM and DM inhibited oxidative DNA damage in H_2_O_2_-induced HepG2 cells.

**Figure 7:**
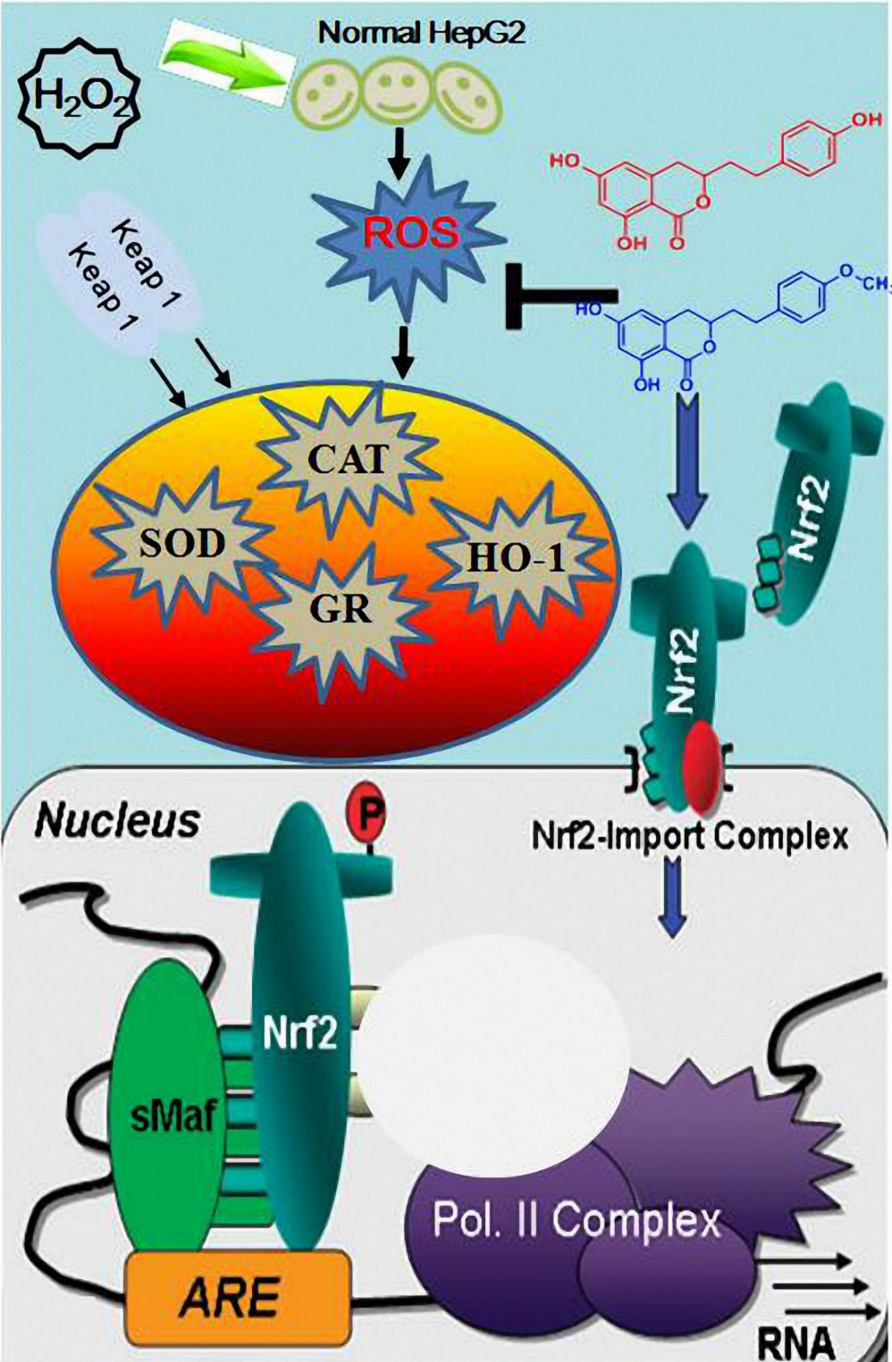
**A schematic representation of the possible mechanism of Keap1-Nrf2/ARE signaling pathway involved in H2O2-induced transformation of HepG2 cells**.

The major strategy of cells to cope with oxidative stress is an increase of the antioxidant response by up-regulating defense enzymes through the activation of Nrf2-ARE. The Nrf2 is the key factor in cell oxidative stress reaction, and through the reaction with ARE, it regulates the expression of a cytoprotective enzyme group. Nrf2, located in the cytoplasm and combined with Keap1 in a physiological state is an important transcription factor for the adjustment of oxidation reaction (Cuadrado, 2015). When body is attacked by factors such as oxygen free radicals and endogenous toxin, the Nrf2 is subsequently dissociated with Keap l, and the half-life of Nrf2 extends obviously, then Nrf2 transposition into the nucleus, and combined with the ARE, induce the expression of a group of cytoprotective enzymes (Figure 7). Nrf2, a major transcription factor, is involved in cellular protection against oxidative stress through ARE-mediated induction of several phase II detoxifying and antioxidant enzymes. The Nrf2-ARE pathway positively regulates expression of antioxidant and detoxification enzymes, including superoxide dismutase (SOD), catalase (CAT), and glutathione reductase (GR) in an effort to reestablish cellular redox homeostasis. It was confirmed that the Nrf2-ARE signal pathway encodes endogenous protection in more than 200 species (Kwak et al., 2003). The endogenous protection can strengthen antioxidant capacity of cells, protecting them from the toxic injury and expressing various biological effects such as anti-tumor, anti-inflammatory and anti-apoptosis. Several structure-activity relationship studies have been previously carried out on the ability of phenolic compounds such as chalcone (Dinkova-Kostova et al., 1998) and curcumin (Thangapazham et al., 2006) to activate the Nrf2-ARE system. The possible mechanism promoting the translocation of Nrf2 to the nuclei is the dissociation of Nrf2 from its intrinsic inhibitor Keap1 through the modification of thiol groups in Keap1 protein. The modified Keap1 protein can thereafter be degraded by the ubiquitin-proteasome pathway, which plays an important role in many cellular processes (Nandi et al., 2006). AM and DM increased the transcriptional activity of Nrf2 and up-regulated several ARE-regulated genes involved in free radical metabolism in an Nrf2-dependent manner (Figure 5). Regarding the possible role of AM and DM in Nrf2 activation, the findings provided a further evidence that AM and DM exerted cytoprotective effects—at least partically—*via* Nrf2-dependent pathways. It is worth of mentioning that AM and DM activated the expression of Nrf2 and ARE-regulated antioxidant genes HO-1. The enzyme HO-1, which catalyzes the first and rate-limiting step of heme degradation, has attracted major attention in recent years. The cytoprotective properties of HO-1 may due to the inhibition of NADPH oxidase activity.

On the other hand, it is widely believed that multiple signaling cascades were implicated in the induction of ARE-dependent phase II detoxifying enzymes. Many studies have declared that several MAPKs, including ERK, JNK, and p38, are involved in regulation of the phosphorylation of Nrf2 and the expression of ARE-mediated phase II and antioxidant genes (Xu et al., 2006). Nevertheless, compared with the ERK and JNK pathways, the p38 pathway plays an opposite role in the induction of detoxifying enzymes (Yu et al., 1999). It is therefore conceivable that the induction of phase II detoxifying enzymes can be either positively or negatively regulated by protein phosphorylation and may involve the differential roles of MAPK family members. For instance, treatment of HepG2 cells with tert-butylhydroquinone (tBHQ) enhanced the induction of NQO1 activity and the activation of ARE reporter by stimulation of the activity of p38 gene (Chen and Kong, 2004). Similarly, tBHQ interfered p38 pathway through the over expression of negative mutant of p38, potentiating the activation of the ARE reporter gene (Yu et al., 2000). Taken all together, the p38 kinase pathway functions should considered as a negative regulator in the ARE-mediated induction of detoxifying enzymes. To further clarify the intracellular signaling pathways, the activation of signaling molecular was examined after AM and DM treatment. We found that the level of active phosphorylated ERK and JNK were significantly higher in AM and DM-treated cells than those in control untreated cells. AM and DM were able to activate JNK and MAP kinases which are pivotal for further activation of Nrf2 in HepG2 cells. Previous studies have also shown that both ERK and JNK are positively involved in ARE-driven gene expression (Keum et al., 2004). Therefore, the antioxidant effect of AM and DM is not only achieved by their non-enzymatic action, but also by the basis of the efficacy of AM and DM to enzymatic actions involved in Nrf2-dependent signaling pathways. It is suggested that MAPKs are important for Nrf2 activation and MAPKs can be induced by AM and DM. Additionally, p38 can also mediate oxidative stress-independent Nrf2-ARE activation. A previous study revealed that activated Nrf2 can induce numerous antioxidant-defense HO-1 genes and suppress adhesion molecule expression by inhibiting phosphorylation/activation of p38 (Huang et al., 2001). Balogun et al. reported that curcumin induced HO-1 expression by promoting the dissociation of the Nrf2-Keap1 complex in a p38-dependent manner (Chen et al., 2012). In another interesting study, Ho et al. also observed that diallyl sulfide activated Nrf2-driven ARE activation and HO-1 was expressed via the p38 pathway (Ho et al., 2011). Supporting this notion, our study showed that p38 was phosphorylated in HepG2 cells after AM and DM treatment, resulting in activating Nrf2/ARE transcriptional activity. In contrast, numerous investigators have also reported that p38 MAPK pathway played a positive role in ARE-dependent phase II detoxifying enzymes (Alam et al., 2000; Kang et al., 2000). In particular, inhibition of p38 activation by a specific p38 inhibitor-SB203580 - potentiated the activation of ARE-dependent reporter gene (Marshall, 1994; Chen et al., 2016; Teng et al., 2016a,b). These contrasting findings could be partially attributed to the pharmacologic inhibitors of p38, suggesting the treatment with these inhibitors suppressed the transcriptional or translational expression of these proteins. Therefore, it should be pointed out that using chemical inhibitors may sometimes be misleading, since chemical inhibitors may not only exclusively inhibit p38 activity, but also to activate functional proteins or other kinases.

In conclusion, here we demonstrated that AM and DM effectively attenuated H_2_O_2_-induced cell damage. The mechanisms underlying this protective effect include: (1) free radicals scavenging activities, (2) activation of Nrf2-driven pathways; (3) inhibition of p38 phosphorylation; (4) activation of ERK, JNK, and MAPK phosphorylation; (5) elevation of the activity of antioxidative enzymes as SOD.

In conclusion, AM and DM treatment results in activation of Nrf2 and MAP kinases and the inhibition of phosphatase activity in hepatocytes, these multiple pathways converge to induce HO-1. Thus, AM and DM can be used at low doses to pharmacologically induce HO-1, although its induction is through generation of non-lethal levels of ROS.

## Author contributions

LC and HT conceived and designed the experiments; LC and HT performed the experiments; LC and JX analyzed the data; LC and JX wrote the paper; KZ, MG, and KS revised the paper.

### Conflict of interest statement

The authors declare that the research was conducted in the absence of any commercial or financial relationships that could be construed as a potential conflict of interest.
